# Intensive Care Nurses' Experiences and Attitudes Towards Family Involvement in Delirium Care: A Qualitative Descriptive Study

**DOI:** 10.1111/nicc.70721

**Published:** 2026-10-08

**Authors:** Cherry Efe Iyiola, Emmanuel Kwame Korsah, Alhassan Sibdow Abukari, Shelley Schmollgruber

**Affiliations:** ^1^ Department of Nursing Education, Faculty of Health Science University of the Witwatersrand Johannesburg South Africa; ^2^ Division of Clinical Associates, Department of Family Medicine and Primary Care, Faculty of Health Science University of the Witwatersrand Johannesburg South Africa; ^3^ Department of Maternal and Child Health, School of Nursing & Midwifery University of Ghana Accra Ghana

**Keywords:** critical care, delirium, family involvement, family‐centred care, intensive care nurses, qualitative research

## Abstract

**Background:**

Delirium is a common neuropsychiatric complication among critically ill patients, particularly those who are mechanically ventilated. Evidence suggests family involvement may improve delirium‐related outcomes in adult intensive care units (ICUs). However, limited evidence exists on intensive care nurses' (ICNs) experiences and attitudes towards family involvement in delirium care in South Africa.

**Aims:**

The study aimed to explore intensive care nurses' experiences and attitudes towards family involvement in the care of mechanically ventilated patients with delirium in adult ICUs.

**Study Design:**

A descriptive qualitative study design was utilised. Four focus group discussions were conducted with 32 ICNs from two adult ICUs at a tertiary academic hospital in Johannesburg between April and August 2022. Participants who had at least 2 years of intensive care experience were purposively sampled. Data were analysed using inductive qualitative content analysis. The study adhered to Consolidated Criteria for Reporting Qualitative Studies guidelines.

**Findings:**

Three categories were identified: emotional burden and psychological demands of delirium care, communication and psychosocial support for families in delirium care and barriers to effective family involvement in delirium care. While recognising the therapeutic value of family engagement, the nurses reported experiencing emotional stress related to family expectations and anxiety. They adopted strategies such as structured communication, empathetic listening and technology‐assisted contact to support families. However, barriers such as time constraints, limited intensive care space, staffing shortages and restrictive policies limited sustained involvement.

**Conclusions:**

This study provides context‐specific insight into how ICNs facilitate family involvement in the care of patients with delirium in a public tertiary ICU. ICNs recognise the therapeutic value of family involvement in the care of patients with delirium and are willing to facilitate engagement. However, systemic and environmental barriers limit consistent implementation. Therefore, strengthening institutional policies and supporting nurses through structured training and team‐based approaches may enhance sustainable family‐centred delirium care.

**Relevance to Clinical Practice:**

This study's findings demonstrate the need for ICNs to adopt structured communication strategies, flexible visitation policies and empathy towards families and their patients in the ICU. Perhaps incorporating family‐centred care into daily intensive care practices could strengthen family involvement in critical care, particularly for patients with delirium.

## Impact Statements

- What is known about the topic
  ○ Delirium is prevalent among mechanically ventilated ICU patients and is associated with increased morbidity and mortality.
  ○ Family involvement has been shown to improve psychological outcomes of delirium patients in the ICU, with nurses playing a central role.
- What this study adds?
  ○ This paper exposed the barriers to family involvement in caring for patients with delirium in the ICU and proposed strategies to navigate the delirium care continuum.
  ○ Adopting family‐centred care into adult ICUs could provide support for patients with delirium and their families to help them navigate the physical and psychosocial burden of care, especially in resource‐limited settings.

## 1 Introduction

Delirium is a common and complex neurocognitive syndrome and a frequently under‐recognised complication of critical illness in the intensive care unit (ICU). Delirium is defined as an acute neurocognitive disorder that develops over a short period of time and is characterised by disturbances in attention, awareness and cognition, with evidence that the disturbance is a direct physiological consequence of an underlying medical condition [1]. In intensive care settings, delirium affects approximately 60%–80% of mechanically ventilated patients [2, 3]. It is associated with prolonged mechanical ventilation, extended ICU and hospital stays, increased morbidity and mortality and long‐term cognitive and psychological sequelae [4, 5]. Despite international guidelines recommending routine delirium screening and multicomponent prevention strategies, delirium continues to be assessed inconsistently and managed variably in clinical practice. The management of delirium presents considerable challenges in ICU settings because it disrupts communication, alters relationships and reshapes everyday care interactions between patients, families and clinicians. These complexities contribute to challenging care processes and may introduce ethical dilemmas in critical care nursing practice [6, 7].

Current evidence‐based guidelines recommend a multicomponent approach to delirium prevention and management [8, 9]. The ABCDEF bundle incorporates family engagement and empowerment (Element F) as a core component of patient‐centred ICU care and recognises families as important partners in delirium prevention and management [8, 9]. Family involvement is increasingly recognised as a key component of delirium prevention and management in the ICU [10, 11]. Contemporary guidelines and care frameworks endorse family engagement as part of person‐centred and humanised care approaches. However, despite its potential clinical benefits, implementing family involvement in delirium care remains complex and context‐dependent. Although the value of family involvement is widely acknowledged, understanding of how intensive care nurses (ICNs) experience, interpret and enact it in practice remains limited. Nurses play a central role in translating policy into practice, yet their perspectives remain underexplored, especially in South African ICUs. Therefore, this study aims to explore ICNs' experiences and attitudes towards family involvement in the care of mechanically ventilated patients with delirium in adult ICUs.

## 2 Background

Intensive care nurses play a critical role in the identification, prevention and management of delirium in the ICU through their continuous bedside presence, assessment and sustained communication with patients and families [4, 12]. They are often responsible for conducting delirium screening, implementing non‐pharmacological interventions and monitoring patients' behavioural and cognitive changes over time. In addition, nurses facilitate communication, provide anticipatory guidance and support family participation in care, particularly during periods of acute confusion and distress [12]. However, evidence indicates that ICU nurses often experience uncertainty and limited confidence in assessing and managing delirium, particularly among sedated and mechanically ventilated patients. These challenges can affect communication, care planning and the overall quality of care provided to patients and their families [4, 5]. Delirium care therefore extends beyond technical competence, requiring nurses to address emotional and relational dimensions such as patient agitation and distress, family concerns and safety concerns.

Family involvement is increasingly incorporated into ICU care practices and delirium prevention and management [10, 11]. Evidence suggests that family‐based interventions may contribute to reducing the incidence and duration of delirium in ICU patients. Interventions such as reorientation, familiar voice and communication, emotional reassurance, cognitive stimulation and physical presence have been used to facilitate family participation in delirium care [11, 13, 14]. Family involvement and engagement are theorised to provide continuity, familiarity and emotional grounding within the technologically intense ICU environment, thereby reducing stress and enhancing patient safety [13].

Despite these benefits, family involvement may introduce emotional and relational complexities for nurses delivering delirium care [4, 5]. For instance, delirium‐related behaviours, including hallucinations, aggression and withdrawal, may be misinterpreted by family members as signs of deterioration and suffering [6, 15]. Nurses must therefore act as interpreters, educators and emotional mediators while maintaining clinical priorities, providing emotional support and ensuring patient safety [4]. These additional responsibilities may increase workload and contribute to care burden and burnout among ICU nurses. In some situations, competing demands may contribute to restrictive practices or ethical tensions, particularly when balancing family involvement with clinical decision‐making and safety concerns [5, 16]. Nurses are often required to negotiate the boundaries of family presence and participation, balancing relational engagement with workload pressures and professional judgement [4, 12].

The implementation of family involvement in delirium care is constrained by structural, cultural and organisational factors [6, 17]. The ICU environment, characterised by high patient acuity, technological demands, rigid routines and infection‐control requirements, may limit opportunities for meaningful family participation and further heighten nurses' concerns regarding safety, disruption and control [5, 17]. These challenges may be particularly relevant in resource‐constrained settings, where staffing shortages and heavy workloads reduce nurses' capacity to facilitate, supervise and support family involvement [4, 18]. Furthermore, inconsistencies in institutional policies regarding visiting practices and family roles contribute to variability in practice and place additional responsibility on individual nurses [4, 15].

Although family involvement in delirium care is endorsed in principle, existing research has predominantly focused on clinical outcomes, intervention effectiveness or implementation feasibility [10, 11, 19]. Nurses' experiences, attitudes and emotional labour regarding family involvement in delirium care have received relatively little attention, particularly in context‐specific qualitative studies. These perspectives are important, as nurses' perceptions may influence their engagement with families, communication practices, boundary setting and the translation of guidelines into practice [4, 5, 15]. There is therefore a need to understand how ICNs experience family involvement in delirium care and how they perceive its role within the organisational and resource constraints of ICU practice, particularly in South Africa. This study aimed to explore ICNs' experiences and attitudes towards family involvement in the care of mechanically ventilated patients with delirium in adult ICUs in South Africa. The findings may inform the development of context‐specific strategies to support ICNs and facilitate appropriate family involvement in delirium care, particularly in low‐resource settings.

### 2.1 Aims

This study aimed to explore ICNs' experiences and attitudes towards family involvement in the care of mechanically ventilated patients with delirium in adult ICUs.

## 3 Design and Methods

### 3.1 Study Design

A qualitative descriptive design was utilised. A qualitative descriptive design aims to provide a rich, straightforward account of participants' experiences in their own words, while remaining close to the data and the clinical reality under study [20]. This approach is particularly well suited to applied nursing research where the focus is on understanding experiences and care practices. The study was reported in accordance with the Consolidated Criteria for Reporting Qualitative Research (COREQ) [21].

### 3.2 Setting

The study was conducted at a tertiary‐level public academic hospital in Johannesburg. The hospital provides secondary, tertiary and quaternary services to patients from Gauteng Province and surrounding regions. Although it is relatively well resourced in terms of infrastructure and specialist clinical services, it operates within a high‐demand public healthcare system characterised by sustained workforce pressure and high patient turnover.

The hospital comprises six adult ICUs with a total capacity of 55 beds. Participants were recruited from a multidisciplinary ICU and a trauma ICU that manage patients with trauma, sepsis, acute respiratory failure, renal failure, multi‐organ dysfunction and complex medical and surgical conditions. The ICUs operate as closed units led by intensivists, with care delivered predominantly by specialist‐trained ICNs. Advanced life‐support modalities, including mechanical ventilation and invasive haemodynamic monitoring, are routinely used, with a nurse–patient ratio of 1:1 for mechanically ventilated patients. Delirium screening and management are routine components of nursing care in these units.

Family visitation follows unit‐level protocols, with flexibility guided by patient acuity, infection‐control considerations and clinical judgement. Care is delivered in a socio‐culturally diverse public‐sector context in which families often play a central supportive role. The site was purposively selected due to its high volume of critically ill patients, frequent exposure to delirium and tertiary academic setting, which provides access to experienced ICU nurses practising in a complex, high‐acuity environment.

### 3.3 Participants and Sample

A purposive criterion sampling strategy was used to recruit ICNs. Eligible participants were registered with the South African Nursing Council, held a post‐basic qualification in critical care nursing, had a minimum of 2 years' clinical experience working in an adult ICU and had direct experience managing patients with delirium and engaging with their families. Participants were also required to be practising in either the multidisciplinary ICU or the trauma ICU at the time of data collection and to be willing to participate in the study. Nurses working in neonatal, paediatric or non‐critical care settings, agency or student nurses, and those in exclusively managerial or educational roles were excluded. These criteria were applied to ensure that participants had sustained, hands‐on experience with delirium care and family involvement in the ICU setting.

### 3.4 Data Collection

Data were collected using a focus group discussion (FGD) guide to facilitate interaction and collective reflection among the participants. The FDG guide (Supplementary file) was developed based on the study objectives and a review of the literature on delirium and family‐centred care in the ICU. Key areas covered in the guide included nurses' experiences with delirium care, attitudes towards family involvement, emotional impact, challenges encountered and suggestions for improving practice. To enhance the relevance and clarity of the FDG guide, it was reviewed by two nursing experts (CI and SS). Before the main data collection, we pre‐tested the guide with four ICNs in a different setting with similar characteristics to assess the clarity, relevance and sequencing of the questions.

Forty‐eight ICNs were approached by the first author (CI), of whom 38 met the inclusion criteria, and 32 consented to participate. We conducted four FGDs with six, eight, eight and 10 participants, respectively. The FGDs were purposively organised into relatively homogeneous groups of ICNs to encourage open discussion among participants with similar clinical contexts. Participants were allocated to groups according to their clinical unit, work shift and availability on the scheduled data collection days. For example, separate FGDs were conducted for nurses working in the multidisciplinary ICU and the trauma ICU, with day‐shift and night‐shift nurses interviewed separately. All FGDs were conducted during participants' scheduled work breaks in a private room within the hospital to minimise disruption to clinical duties and patient care. This approach facilitated discussion among nurses who shared similar work environments and experiences while accommodating the operational demands of the ICU.

The FGDs were facilitated by the first author (CI), while a trained researcher with experience in qualitative research served as an observer and recorded field notes. The discussions lasted 45–60 min and were audio‐recorded with participants' consent. Field notes captured non‐verbal cues, group interactions and contextual observations to complement the audio recordings. Discussions were held in private rooms to ensure confidentiality and minimise interruptions. Data were collected between April and August 2022. In this study, the sample size was guided by the principle of data saturation rather than a predetermined numerical target, with recruitment continuing until no substantively new themes or categories emerged.

Reflexivity was maintained throughout the study to ensure rigour. The first author (CI) is a trained qualitative researcher and registered critical care nurse with ICU experience. A reflexive journal and reflective field notes were maintained during data collection by the first author (CI) to critically examine personal assumptions and their potential influence on data collection and interpretation. The semi‐structured FGD interview guide was used consistently across all focus groups, and care was taken to avoid leading questions. After each focus group discussion, the first author (CI) and three other experienced qualitative researchers (SS, EKK and ASA) met to review the data and inform subsequent FGDs. To support the credibility and accuracy of the data, the transcripts were returned to participants for review, thereby contributing to the rigour of the study.

### 3.5 Data Analysis

Data were analysed using conventional qualitative content analysis as described by Erlingsson and Brysiewicz [22]. This inductive analytic approach allowed categories and themes to emerge directly from participants' accounts while remaining close to the manifest and latent content of the data. The researchers conducted the analysis collaboratively.

All focus group transcripts were read repeatedly to achieve immersion in the data and a holistic understanding of the dataset. Meaning units relevant to the study aim were identified and extracted. The first author (CI) conducted initial coding, which a nursing professor (SS) then reviewed and co‐coded. The research team developed and used a codebook containing definitions to conduct the content analysis across all transcripts. The research team compared the codes within and across transcripts to identify similarities and differences. Disagreements in codes and sub‐categories were settled by a nursing professor (SS) with a strong qualitative research background. The codes and subcategories were discussed and resolved with the input from a nursing professor (SS) with extensive qualitative experience. The codes were then abstracted into broad categories that represented the data. During the content analysis, the authors had regular meetings and discussions to build consensus. These iterative analytical processes were documented in an audit trail to maintain methodological rigour.

### 3.6 Ethical Considerations

Ethical approval was granted by the University Human Research Ethics Committee (Approval No: M210533; 15/12/2021) and the study hospital (Approval Number: GP_202107_011; 30 September 2021). The researcher obtained written informed consent from all the participants before participation and audio‐recording of the FGDs. Each participant signed a consent form confirming their willingness to participate in the study before discussions commenced. The study maintained confidentiality and anonymity, and participants were informed of their right to withdraw at any stage. Participants were also made aware of the inherent limitations of confidentiality associated with focus group research.

### 3.7 Trustworthiness

Trustworthiness was ensured through credibility, dependability, confirmability and transferability, consistent with established standards for qualitative nursing research [23]. Credibility was supported through prolonged engagement with the data, interactive FGDs and participant validation via end‐of‐session summaries. It was further enhanced through peer debriefing during data analysis. Although the first author (CI) independently facilitated all focus group discussions, the study supervisor (SS) and two additional researchers (EKK, ASA), experienced in qualitative research, subsequently reviewed and discussed the coding process, emerging categories and theme development. Differences in interpretation were resolved through discussion until consensus was reached.

Dependability was enhanced through a transparent and systematic analytic procedure, with detailed documentation of methodological decisions related to sampling, data collection, coding, category development and theme generation. An audit trail was maintained throughout the study, enabling traceability of analytic decisions and enhancing the study's methodological coherence and dependability. Confirmability was addressed through collaborative analysis, reflexive field notes and peer debriefing, which helped minimise the influence of individual assumptions. Transferability was supported through rich descriptions of the study setting, participants and clinical context, enabling readers to assess the applicability of the findings to comparable ICU environments.

## 4 Findings

Thirty‐two ICNs participated in this study, including 20 from a multidisciplinary ICU and 12 from a trauma ICU. The sample comprised 21 females and 11 males aged 24–60 years. All participants held critical care qualifications and had more than 2 years of ICU experience. Nineteen participants had between 2 and 10 years of ICU experience, while 13 had more than 10 years. Most participants held diplomas or advanced diplomas (*n* = 23); five held undergraduate degrees; and four held master's degrees.

Through inductive content analysis, three categories were identified: Emotional Burden and psychological demands of Delirium care, Communication and psychosocial support for families in delirium care and Barriers to Effective Family Involvement in delirium care. These categories illuminate the complexity of ICU nursing practice in relation to family involvement in delirium care. Table 1 presents the categories, subcategories and selected example codes derived from the data.

**TABLE 1:** Categories, subcategories and example codes.

Categories	Subcategories	Example codes
Emotional burden and psychological demands of delirium care	Stress and anxiety	Family distress increases nurse anxiety, emotional intensity affects clinical concentration, repeated reassurance demands, tension between compassionate responsiveness and task‐oriented care
	Emotional exhaustion	Feeling emotionally drained at the end of the shift, cumulative relational fatigue, balancing patient care and family support, psychological cost of caring
	Managing family expectations	Families expect rapid improvement, tension regarding ‘doing enough’, explaining uncertain prognosis, mediating hope and clinical reality
Communication and psychosocial support for families in delirium care	Sharing information and updates	Explaining clinical conditions, repetition of information, translating medical terminology, promoting understanding and involvement
	Emotional support through communication	Active listening, demonstrating empathy, providing reassurance, regular updates, addressing family concerns
	Empathy and presence	Being emotionally available, offering comfort, therapeutic presence and sustained relational engagement
Barriers to effective family involvement in delirium care	Time constraints	Fast‐paced ICU environment, prioritisation of clinical tasks, limited time for discussion, communication takes a secondary role
	Limited family space	Lack of waiting areas, restricted bedside presence, environmental constraints, absence of designated family zones
	Staffing shortages	Understaffing, increased workload, reduced time for communication, stress from short‐staffed shifts
	Hospital policies	Visiting hour restrictions, no overnight stays, institutional limitations on family presence, policy‐driven access control

### 4.1 Category 1: Emotional Burden and Psychological Demands of Delirium Care

This category captures the emotional and psychological challenges ICNs experienced when caring for mechanically ventilated patients with delirium and their families. Three sub‐categories emerged from the data to support this category: stress and anxiety, emotional exhaustion and managing family expectations.

#### 4.1.1 Stress and Anxiety

Participants described increased stress when caring for patients experiencing delirium. This stress was associated with family distress, repeated requests for reassurance, frequent questioning and the emotional demands of simultaneously caring for patients with delirium and supporting their families. Participants described these demands as affecting their ability to maintain concentration during clinical care. The following extracts illuminate these findings:

I feel anxious when the family is upset or confused; it adds to my stress (P6).

The emotional intensity of the situation makes it harder to focus on clinical care, especially when the family is worried all the time (P7).

It's stressful when the family asks so many questions, and you just don't have enough time to answer them all (P15).

Because when the patient starts to wake up and sees these machines… people that they don't know, it's like strangers to them; they become so confused. … (P3)

#### 4.1.2 Emotional Exhaustion

Beyond immediate stress, participants described cumulative emotional exhaustion associated with sustained relational engagement with families of delirium patients. They attributed it to the ongoing demands of reorienting patients, balancing family needs and caring for patients experiencing delirium.

There's always this emotional burden, because you want to do your best, but it's so emotionally draining (P12).

At the end of my shift, I feel exhausted from trying to manage patient care and be there for families (P5).

I often leave work feeling emotionally drained, especially after dealing with worried families all day (P9).

The other thing would be to reorient them… the environment is unfamiliar, the faces are unfamiliar, and maybe some medications make them delirious. Sometimes they don't know whether it's day or night (P7).

#### 4.1.3 Managing Family Expectations

Another aspect of emotional burden involved managing family expectations that did not always align with the clinical realities of delirium care. Nurses described families' hopes for rapid improvement, certainty or a cure, particularly in the context of fluctuating and unpredictable clinical trajectories as contributing to emotional strain. Participants also described the challenges of acting as intermediaries between clinical uncertainty and family expectations. In this context, educating families about the patient's condition was seen as important for supporting their understanding and involvement.

The family always asks if we're doing enough for the patient, and it creates tension (P18).

Sometimes, I feel the family thinks we can just ‘fix’ the patient, and when things aren't improving quickly, they get frustrated (P2).

It's hard to explain to families that their loved one's condition isn't always going to improve in a way they expect (P10).

Overall … the entire thing is educating them. You must inform the family first about the condition… explain and counsel them about the condition, and that's when they will start supporting the patient. (P19)

### 4.2 Category 2: Communication and Psychosocial Support for Families in Delirium Care

Despite the emotional strain articulated above, participants consistently affirmed the importance of communication and providing psychosocial support for families in delirium care. ICNs described providing this support through information sharing and regular updates, emotional support, empathy and presence.

#### 4.2.1 Sharing Information and Updates

The participants described information sharing as foundational to family involvement. They provided clear explanations, repeating information where necessary, and translating complex clinical information into understandable language to reduce anxiety and support family involvement in care. Additionally, the ICNs emphasised that communication was particularly important when caring for patients experiencing delirium, as families often required repeated explanations regarding the patient's confusion, altered behaviour and fluctuating condition.

*If* families understand delirium, they can be more involved and less anxious (P16).

It's helpful when I can explain what delirium is and why it's happening; families seem to understand more and feel more involved (P4).

We always explain to the family what's going on with the patient, but it's important to repeat it often (P13).

Well, whenever there's confusion with the patients, we know that they have delirium and we update the family… you can allow them to come in anytime… because it also helps (P19).

#### 4.2.2 Emotional Support Through Communication

ICNs utilised communication as a tool to provide emotional support for families with delirium patients in the ICU. This was achieved through active listening, reassurance and regular updates on the patient's condition to address the family's concerns. The following extracts illustrate these experiences.

I try to keep the family updated regularly about their loved one's condition (P9).

Listening to the family's concerns and showing them, empathy helps ease their anxiety (P17).

The families feel better when we listen to them, even if we can't offer them immediate solutions (P8).

Overall … the entire thing is educating them. You must inform the family first about the condition… explain and counsel them about delirium, and that's when they will start supporting the patient (P19).

#### 4.2.3 Empathy and Presence

The participants described empathy and presence as important components of psychosocial support for families. They demonstrated this support by being emotionally available, offering comfort, remaining physically present and listening to family concerns.

I listen to the family's concerns and offer emotional support, especially when they feel helpless (P1).

It's important to be there for the family; sometimes they just need someone to listen (P3).

I try to reassure the family by showing them empathy and explaining that we're doing everything we can (P6).

…So just having a familiar face can improve the way they feel, allay anxiety and do your job so much better because of this understanding (P16).

### 4.3 Category 3: Barriers to Effective Family Involvement in Delirium Care

This category describes the organisational and structural constraints that limited nurses' ability to involve families in delirium care. These barriers included: time constraints, limited family space, staffing shortages and hospital policies.

#### 4.3.1 Time Constraints

Time constraints emerged as a perceived barrier to effective family involvement in delirium care. The ICU environment was described as fast‐paced, task‐driven and prioritised based on acute clinical demands. Under such conditions, participants reported that meaningful family involvement could be deprioritised in favour of immediate patient‐care needs.

There's simply not enough time to sit and talk with families; the ICU environment is just too fast‐paced (P19).

I have to move quickly from one task to another, and family communication often takes a back seat (P14).

Time pressure is a huge challenge; I want to spend more time with the family, but there's just not enough time (P16).

Because sometimes the patients are so restless, you end up concentrating on stabilising the patient first, and there isn't enough time to explain everything to the family (P4).

#### 4.3.2 Limited Family Space

Participants described the ICU's physical layout as a potential barrier to effective family involvement in delirium care. They reported limited space within and around the ICU for family waiting, proximity to the patient and opportunities for comfort and interaction. These constraints were perceived to limit families' ability to stay close to and participate in their relatives' care.

Family members often don't have anywhere comfortable to stay while waiting (P3).

The lack of space for family members makes it hard for them to stay close to the patient (P2).

It's difficult for families to be involved when they're not allowed to stay near their loved one due to space constraints (P11).

#### 4.3.3 Staffing Shortages

Participants also described inadequate staffing as an important obstacle to family involvement in delirium care. Staff shortages increased nurses' workloads and reduced time available for patient care, communication with families and emotional support.

I sometimes feel I can't give the family enough attention because of staff shortages (P11).

With fewer nurses, it's difficult to spend enough time communicating with families (P7).

When we're short‐staffed, I can't focus on the family as much, which adds stress (P4).

#### 4.3.4 Hospital Policies

Institutional policies were also identified as obstacles to family‐centred practices. Participants specifically described visiting restrictions and limitations on overnight hospital stays as constraining consistent family presence.

Hospital policies prevent families from staying overnight, which makes it hard for them to be involved as much as they'd like (P4).

The visiting hours are too limited, which prevents families from having consistent involvement (P15).

Policies that limit family presence during certain times make it harder to keep families informed and involved (P8).

## 5 Discussion

This study was designed to explore ICNs' experiences and attitudes towards family involvement in the care of patients with delirium in a South African adult ICU. We found that the ICNs are constrained in their ability to involve family in the ICU due to low staffing levels, visiting policies and limited space. These constraints increase the burden of care in the ICU, which includes both the emotional and physical challenges of managing patients, especially those with delirium, as well as supporting their families. ICNs recognise family involvement and presence as a significant component of care in the ICU, especially when managing their patients' and their families' psychosocial needs. As such, they adopted context‐specific interventions, including effective, empathetic information sharing and the deployment of technology to manage the situation. These findings demonstrate the critical value of family presence and involvement in the ICU and re‐emphasise the need for institutional support to help ICNs and families navigate the barriers to improve quality health care in low‐resource settings.

Working in the ICU is associated with a significant emotional burden, particularly when caring for families affected by neurocognitive disorders like delirium [5, 24, 25]. The management of patients experiencing delirium often leads to heightened anxiety and uncertainty about outcomes, primarily due to the continuous changes in patients' level of consciousness. Research indicates that delirium increases emotional strain, which can negatively affect nurse‐family therapeutic relationships and behavioural and communication patterns [24, 26]. Intensive care nurses face a dual challenge of delivering advanced nursing care while also addressing the psychosocial needs of patients and their families. This dual responsibility contributes to the emotional burden and burnout among ICNs. The literature demonstrates that the complex demands of ICU care induce stress among ICNs, potentially compromising effective communication between nurses and families [16, 27]. These challenges are more pronounced when caring for delirious patients in the ICU, as the condition exacerbates emotional stress and anxiety, further hindering therapeutic communication [5, 28]. These ongoing emotional demands highlight the need for nurses to embrace resilience and family‐centred care principles to support patients and families in the ICU. Therefore, to maximise nurses' ability to navigate these emotional constraints and improve the quality of care in the ICU, the study proposes providing emotional support systems, particularly communication training, counselling services and debriefing sessions.

Furthermore, the study findings demonstrate the therapeutic value of communication and psychosocial support as an important part of family involvement in managing patients with delirium in the ICU. Nurses recognise the therapeutic functions of the family, including orientation and psychosocial stability, when they are actively engaged in the care processes. Earlier research has shown that the presence of family members at the bedside of patients with delirium in the ICU helps reduce confusion by offering orientation, emotional reassurance and enhances the patient's cognitive functioning [10, 29, 30]. Additionally, family presence in the ICU fosters improved communication, partnerships and collaboration, which in turn promotes family‐centred care and enhances the quality of care in the ICU [14, 31]. Moreover, allowing family involvement in the ICU has proved effective in reducing family stress and anxiety arising from the complexities of the ICU environment, thereby supporting the cognitive functioning of both the patient and their families [29, 32]. These practices also contribute to improved patient and family education, facilitating continuity of care after discharge from the ICU [11, 19]. To bridge the gap between clinical care and social support within a holistic care framework, the integration of families into delirium management is essential. As a result, nurses could adopt structured, context‐specific family involvement interventions that encompass effective, sympathetic communication, information sharing and active participation to maximise the therapeutic value that families can provide in the care of patients with delirium in the ICU.

Despite the acknowledged benefits of family involvement in the management of patients with delirium in the ICU, various systemic and environmental barriers continue to limit family engagement [33, 34, 35]. The absence of guidelines and logistical constraints compels nurses to prioritise clinical care over activities that promote family participation [35]. An increased workload coupled with insufficient staffing further exacerbates this tendency within the ICU [33, 36]. Additionally, most ICUs operate with strict entry protocols and implement fragmented family‐centred practices, which ultimately impede comprehensive family involvement in the care of their loved ones [33]. In practice, these barriers hinder opportunities to enhance family involvement in the care of patients with delirium and undermine the sustainability of family‐centred care, which is essential for achieving quality healthcare outcomes. Consequently, the interaction between systemic and contextual barriers affects family participation in the care of their loved ones with delirium. To foster a conducive environment and maximise family involvement in delirium management, institutions need to prioritise adequate resources, including training specialised nurses, developing flexible visitation protocols and developing delirium care guidelines.

### 5.1 Strengths and Limitations

The strength of this study lies in the use of a descriptive qualitative design to present the contextual perspectives of ICUs regarding delirium management. The design has also permitted the presentation of the intricate emotional, organisational and relational aspects of family involvement in delirium management. The researchers adhered to the COREQ guidelines and used inductive content analysis to enhance the study's transparency and rigour. Despite these strengths, there are some limitations worth noting. For instance, conducting the study in only two ICUs within a single metropolitan area could compromise its transferability. Perhaps the use of FGDs has compromised the disclosure of sensitive experiences from the participants. For example, participants may have been reluctant to disclose sensitive experiences, such as conflicts with family members, emotional distress arising from the management of patients with delirium or challenges encountered when involving families in caring for patients with delirium.

Furthermore, the inclusion of diverse participants, including families and doctors, could have triangulated the results, enriching the findings.

### 5.2 Implication for Practice, Policy and Research

Our findings demonstrate the value of integrating family in the management of delirium in the ICU. Practically, families can be involved in the ICU through family‐centred care interventions, including family acceptance, reorientation to the ICU environment, flexible visiting policies and providing psychosocial care. These interventions require institutional support in staff training, adequate staffing and logistics within the ICU. This support will enhance staff capacity and reduce the workload in the ICU, leading to improved care outcomes.

Additionally, ICUs should adopt empathetic communication models to share information with families [37]. Some methods of empathetic communication include providing clear and honest explanations about delirium and its fluctuating nature, actively listening to families' concerns, acknowledging their emotional distress, offering reassurance during episodes of confusion or agitation and encouraging families to participate in simple reorientation strategies. These approaches help families to better understand delirium and their roles, become active partners and feel comfortable sharing observations and concerns. Subsequently, it reduces family anxiety and strengthens nurse–family partnerships in patient care. Furthermore, these interventions can be supplemented by providing counselling services to help manage the emotional burden experienced by both nurses and their families in the ICU. Future research could evaluate these research findings in a larger sample to develop context‐specific guidelines for family participation in delirium management that could address these concerns in low‐resource settings.

## 6 Conclusion

This qualitative study has highlighted the emotional burden, therapeutic value, and systemic barriers associated with family involvement in the care of delirium patients in the ICU. To navigate the burden of care for patients with delirium in the ICU, the integration of family‐centred care in a resource‐constrained setting is proposed. Future research could engage diverse participants to develop context‐specific family participation guidelines for delirium management in low‐resource settings.

## Author Contributions

**Cherry Efe Iyiola:** conceptualization, data curation, formal analysis, methodology, resources, project administration, visualization, writing – original draft, writing – review and editing. **Emmanuel Kwame Korsah:** conceptualization, methodology, supervision, validation, visualization, writing – original draft, writing – review and editing. **Alhassan Sibdow Abukari:** data curation, formal analysis, methodology, validation, visualization, writing – original draft, writing – review and editing. **Shelley Schmollgruber:** conceptualization, data curation, formal analysis, methodology, supervision, validation, visualization, writing – original draft, writing – review and editing.

## Funding

The authors have nothing to report.

## Ethics Statement

Ethics approval was obtained from the Human Research Ethics Committee (Medical) of the University of the Witwatersrand (Protocol Number: M210533, 15 December 2021) and the Johannesburg academic hospital (Ethic approval number: GP_202107_011, 30 September 2021).

## Consent

Before beginning study procedures, participants signed an informed consent form, a data privacy agreement and consented to the audio‐recording of the interview.

## Conflicts of Interest

The authors declare no conflicts of interest.

## Supporting information


**Data S1:** Supporting Information.

## Data Availability

The data that support the findings of this study are available upon request from the corresponding author. The data are not publicly available due to privacy or ethical restrictions.
