# Supplementary material for: Intensive Care Nurses' Experiences and Attitudes Towards Family Involvement in Delirium Care: A Qualitative Descriptive Study
Source: Nurs Crit Care. 2026 Oct 8;31(6):e70721. doi: 10.1111/nicc.70721 (PMC13646848; doi:10.1111/nicc.70721)
Supplement: Supplementary file 1 [file NICC-31-0-s001.docx]

**Supplementary File**

**FOCUS GROUP DISCUSSION INTERVIEW GUIDE**

Can you share your experiences of involving family members in the care of ICU patients with delirium?

***Probes***

- How did you feel involving the family in the care of the patient?
- How has family involvement affected you emotionally or professionally?
- How do you support and communicate with families during episodes of delirium>
- What specific challenges have you experienced when involving the families in delirium care?
- What strategies could improve family involvement in delirium care?
